# Slow-Release Pharmaceutical Implants in Ecotoxicology: Validating Functionality across Exposure Scenarios

**DOI:** 10.1021/acsenvironau.4c00056

**Published:** 2024-11-25

**Authors:** Michael G. Bertram, Jack A. Brand, Eli S. J. Thoré, Daniel Cerveny, Erin S. McCallum, Marcus Michelangeli, Jake M. Martin, Jerker Fick, Tomas Brodin

**Affiliations:** † Department of Wildlife, Fish, and Environmental Studies, Swedish University of Agricultural Sciences, Umeå SE-907 36, Sweden; ‡ Department of Zoology, Stockholm University, Stockholm 114 18, Sweden; § School of Biological Sciences, Monash University, Melbourne, 3800, Australia; ∥ Institute of Zoology, Zoological Society of London, London NW1 4RY, United Kingdom; ⊥ TRANSfarm - Science, Engineering, & Technology Group, KU Leuven, Lovenjoel 3360, Belgium; # Laboratory of Adaptive Biodynamics, Research Unit of Environmental and Evolutionary Biology, Institute of Life, Earth, and Environment, University of Namur, Namur 5000, Belgium; 7 Faculty of Fisheries and Protection of Waters, South Bohemian Research Center of Aquaculture and Biodiversity of Hydrocenoses, University of South Bohemia in Ceske Budejovice, Vodnany 389 25, Czech Republic; 8 Australian Rivers Institute, Griffith University, Nathan 4111, Australia; 9 School of Life and Environmental Sciences, Deakin University, Waurn Ponds 3216, Australia; 10 Department of Chemistry, Umeå University, Umeå 907 36, Sweden

**Keywords:** behavior, contaminant, dose, drug, fish, salmon

## Abstract

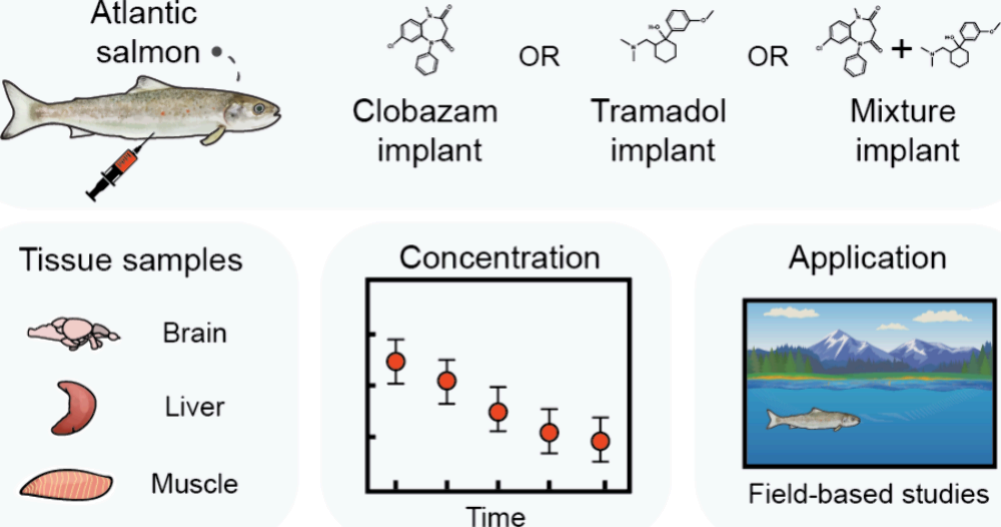

Pharmaceutical contaminants have spread in natural environments across the globe, endangering biodiversity, ecosystem functioning, and public health. Research on the environmental impacts of pharmaceuticals is growing rapidly, although a majority of studies are still conducted under controlled laboratory conditions. As such, there is an urgent need to understand the impacts of pharmaceutical exposures on wildlife in complex, real-world scenarios. Here, we validate the performance of slow-release pharmaceutical implants—a recently developed tool in field-based ecotoxicology that allows for the controlled chemical dosing of free-roaming aquatic species—in terms of the accumulation and distribution of pharmaceuticals of interest in tissues. Across two years, we directly exposed 256 Atlantic salmon (*Salmo salar*) smolts to one of four pharmaceutical treatments: clobazam (50 μg g^–1^ of implant), tramadol (50 μg g^–1^), clobazam and tramadol (50 μg g^–1^ of each), and control (0 μg g^–1^). Fish dosed with slow-release implants containing clobazam or tramadol, or their mixture, accumulated these pharmaceuticals in all of the sampled tissues: brain, liver, and muscle. Concentrations of both pharmaceuticals peaked in all tissues at 1 day post-implantation, before reaching relatively stable, slowly declining concentrations for the remainder of the 30-day sampling period. Generally, the highest concentrations of clobazam and tramadol were detected in the liver, followed by the brain and then muscle, with observed concentrations of each pharmaceutical being higher in the single-exposure treatments relative to the mixture exposure. Taken together, our findings underscore the utility of slow-release implants as a tool in field-based ecotoxicology, which is an urgent research priority given the current lack of knowledge on the real-world impacts of pharmaceuticals on wildlife.

## Introduction

Ecosystems around the globe are increasingly contaminated with active pharmaceutical ingredients (APIs).^1,2^ Research conducted over the last three decades has demonstrated that exposure to APIs can alter a wide range of fundamental processes in organisms, from development^3^ to reproduction,^4^ metabolism and physiology,^5^ and morphology.^6^ Moreover, a rapidly growing body of research has shown that API pollution can alter a wide array of key behaviors in animals,^7,8^ with potentially dire implications for individual fitness and population persistence.^9^

Despite recent advances in studying the behavioral impacts of pharmaceutical exposure—and exposure to chemical contaminants more generally—the vast majority of studies in this area have been conducted under controlled, often oversimplified, laboratory conditions.^8^ This is true even though organisms in the wild live in complex multistressor environments that vary over time and space. Hence, although laboratory-based studies are undoubtedly crucial in understanding the impacts of API exposure on animal behavior, including identifying specific molecular mechanisms underpinning observed behavioral changes, there is an urgent need for more behavioral ecotoxicology research conducted under natural and seminatural conditions.^8^ This is vital because environmental protection efforts are focused on the health of populations, as opposed to individuals, meaning that studies demonstrating effects of pollutants on the behavior of animal populations in the wild are necessary to increase the adoption of behavioral endpoints into risk assessment and regulatory decision making.^10,11^

A wide array of recently developed tools and techniques now facilitate studying the impacts of APIs on behavioral parameters in the wild with unprecedented experimental design complexity, detail, and accuracy.^8^ One of the most promising approaches in aquatic environments is the use of remote-sensing technologies like acoustic telemetry, a tracking technology that facilitates detailed study of the movement of free-roaming animals.^12−14^ Concurrently, ongoing advancements in biologging technologies offer a wide array of small physiological and behavioral sensors that can capture data ranging from an animal’s heart rate, body temperature, and acceleration to intricate details of foraging, social and spawning behaviors, and even predation events.^15,16^ Animal-tracking and biologging approaches enable data collection on behavioral processes that were previously difficult or impossible to measure in the wild, which is also the case for nonbehavioral processes—e.g. potential impacts of contaminant exposure on the heart rate and/or body temperature of free-roaming animals.

The overlap between telemetry and biologging approaches with ecotoxicology research has, to date, been limited.^8,17^ Moreover, research that has been done has conventionally been restricted in terms of experimental design options. For instance, studies investigating the impacts of API exposure on fish in natural or seminatural systems have typically involved exposing study organisms to APIs in the laboratory before release^18^ or dosing an entire aquatic ecosystem with an API to ensure exposure throughout the study period.^19^ As such, fish were not continually exposed throughout the entire study duration or an entire aquatic ecosystem was contaminated with an API, respectively.

Future research is poised to combine acoustic telemetry and biologging with emerging methods of remote contaminant exposure to gain valuable insights into the real-world behavioral and physiological impacts of API pollution. In this regard, one particularly promising, recently developed approach is the use of slow-release pharmaceutical implants.^20,21^ These low-cost, fat-based, slow-release implants facilitate continuously exposing tracked animals released into seminatural and natural aquatic environments over days to months. Moreover, the use of slow-release implants circumvents the existing limitations of fish depurating APIs throughout the behavior-tracking period (after being exposed in the laboratory and released into the wild) or having to expose an entire ecosystem to one or more APIs throughout a study. At present, however, slow-release pharmaceutical implants have not been validated for use across a broad range of species, contaminants, and exposure scenarios (e.g., individual chemical versus mixture exposure). Such validation is a vital foundation for future ecotoxicology studies investigating the impacts of APIs on aquatic species.

Here, in a large-scale, laboratory-based study performed across two years, we exposed two-year-old Atlantic salmon (*Salmo salar*) smolts to one of four pharmaceutical treatments via slow-release implants: clobazam (50 μg g^–1^ of implant), tramadol (50 μg g^–1^), clobazam and tramadol (50 μg g^–1^ of each), and control (0 μg g^–1^). Importantly, benzodiazepine (e.g., clobazam) and opioid (e.g., tramadol) drugs are routinely detected in the environment,^1,22^ can have adverse chemical interactions when prescribed together to human patients,^23^ and may be expected to negatively affect wildlife when exposed simultaneously. Brain, liver, and muscle tissues were then sampled from smolts at regular intervals over a 30-day sampling period, replicated across years. We present the accumulation of both drugs and their mixture in smolt tissues over time, as well as discussing the implications of our results for future experiments employing slow-release pharmaceutical implants in ecotoxicology.

## Materials and Methods

To examine the accumulation and distribution of clobazam and tramadol in fish tissues, Atlantic salmon smolts received slow-release implants in 2020 and 2021. We randomly selected a total of 256 two-year-old smolts (128 per year, mean body mass 68.2 g ± 29.6 g) from the hatchery stocks of the Fisheries Research Station of SLU Aqua (Älvkarleby, Sweden), which is also where the experiment was conducted. Smolts were divided into four treatment groups (32 fish per treatment each year): clobazam, tramadol, mixture, and control. Fish were kept in large flow-through tanks (1 m length × 1 m width × 0.3 m height; ∼300 L; 2 tanks per treatment; 16 fish per tank), receiving freshwater from the River Dal, and were not fed during the exposure period, in line with standard husbandry practices for premigration Atlantic salmon smolts. Fish were kept in flowing water directly from the River Dal to simulate as closely as possible natural water conditions, such as water chemistry and temperature (mean ± *SE* water temperature during the study period: 2020 = 10.06 ± 0.28 °C; 2021 = 12.78 ± 0.47 °C), as well as being kept under ambient lighting (∼13:11 h light:dark). Fish were checked daily throughout the experimental period, and all handling procedures were approved by the Swedish Board of Agriculture (permit numbers: Dnr A.18.15 and Dnr 5.8.18).

The preparation of implants followed published protocols.^20^ Clobazam (CAS: 22316-47-8, ≥98% purity) and/or tramadol hydrochloride (CAS: 36282-47-0, ≥99% purity), purchased from Merck (Darmstadt, Germany), were dissolved in liquid coconut oil (CO, Kung’s Markatta Virgin Coconut Oil) at 30 °C. To ensure thorough mixing, the compounds were continuously stirred in the coconut oil for 10 min and sonicated for 15 min in an ultrasound bath. In total, 100 g of implant was prepared for each treatment by adding 5 mg of either tramadol, clobazam, or both compounds to reach the nominal concentration of 50 μg per g of implant. The resulting solutions were then injected intraperitoneally into smolts (anaesthetized using 0.15 μg L^–1^ MS-222, CAS: 886-86-2, ≥98% purity; Merck) with a blunted 18-gauge needle at a dose of 5 μL of implant per g of body mass. Given that the density of the fat-based carrier ranges between 0.903 and 0.921 g per mL, 5 μL of implant corresponds with approximately 0.23 μg of (each) pharmaceutical being injected into the fish per gram of its body weight. The implant solidifies upon administration, exposing fish at a concentration of 50 μg of clobazam per g of implant, 50 μg of tramadol per g of implant, 50 μg of clobazam + 50 μg of tramadol per g of implant (mixture), or 0 μg of pharmaceutical per g of implant (control). These dosages were selected to approximate the levels of clobazam and tramadol to which fish are exposed in contaminated natural systems. More specifically, clobazam and other benzodiazepines with the same mechanism of action are frequently detected in wastewater-impacted aquatic ecosystems around the globe, which includes the native distribution of Atlantic salmon.^1,22,24−28^ This is also the case for tramadol and other opioid pharmaceuticals.^27,29−33^ As such, tissue concentrations in our study were specifically targeted to be representative of levels detected in fish from highly contaminated systems worldwide.^34−36^

**Figure 1:**
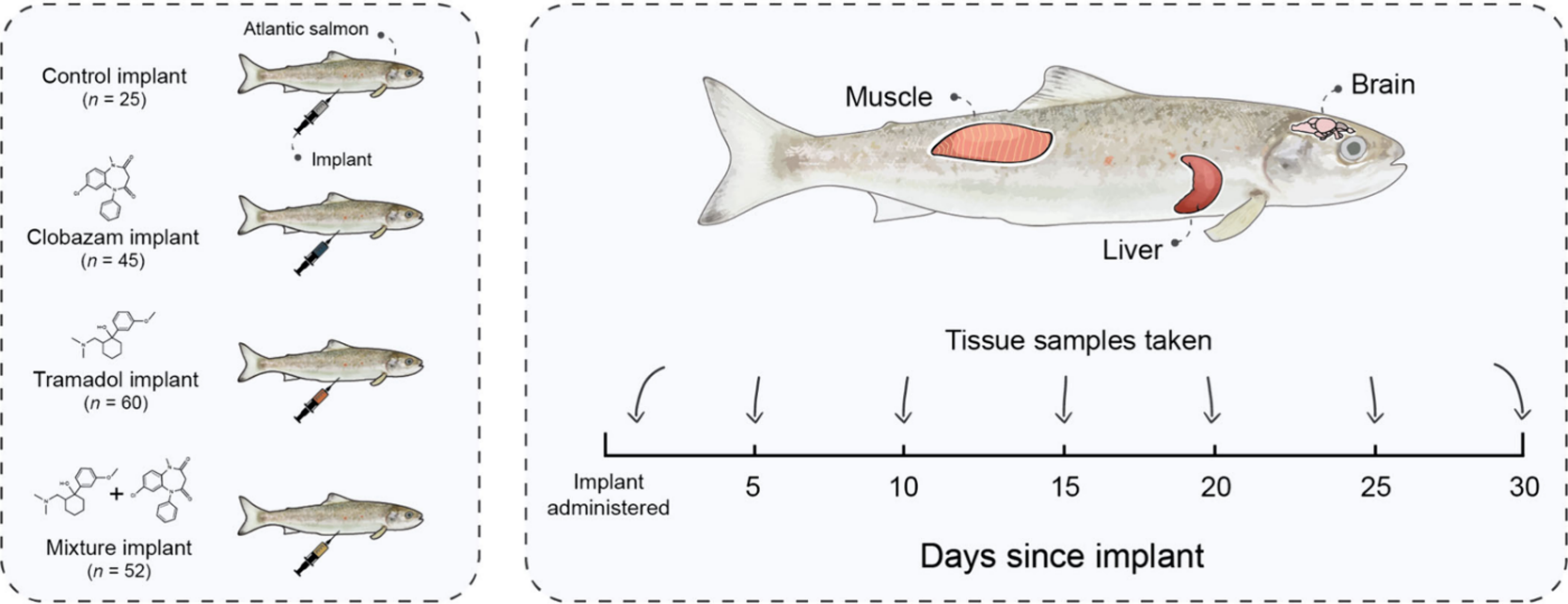
Experimental overview. Sample sizes represent the number of fish used in the analysis of tissue-concentration data (control group not sampled on days 5 and 10 post-implantation). Atlantic salmon smolt photo insert credit: Jörgen Wiklund.

At seven time points post-implantation (24 h, 5 d, 10 d, 15 d, 20 d, 25 d, and 30 d; Figure 1), two randomly selected fish per treatment group per tank (four fish per treatment) were euthanized with MS-222 (0.4 g L^–1^) and frozen at −20 °C. Later, frozen fish were thawed for 30 min and dissected to obtain brain, liver, and muscle tissues (0.11 ± 0.01 g of tissue per sample) for clobazam and tramadol concentration analysis through liquid chromatography–tandem mass spectrometry (see the Supporting Information). Tissues from five time points post-implantation (24 h, 15 d, 20 d, 25 d, and 30 d) were also collected from fish in the control group, which were analyzed to confirm the absence of clobazam and tramadol (all control samples were below the limit of quantification [LOQ] for both clobazam and tramadol). Tissue samples from the clobazam, tramadol, or mixture treatment groups that were < LOQ were given half the relevant LOQ (mean LOQ ± *SE*; clobazam = 0.35 ± 0.02 ng g^–1^; tramadol = 0.17 ± 0.01 ng g^–1^) for inclusion in mean concentration calculations, in line with previous research.^20^

### Statistical Analysis

Prior to analysis, data cleaning (e.g., removing samples where the implant was actively touching the target tissue during sampling, samples damaged during preparation) was conducted to ensure high-quality data and resulted in tissues from a total of 182 fish being included in the analysis. Concentration data were analyzed using Bayesian generalized linear models with an exponential distribution (log link) in the *brms*(37) package within the R statistical environment.^38^ Post hoc comparisons were performed using the *emmeans*(39) and *modelbased* packages from the *easystats* suite^40^ and are reported as ratios of geometric means. We report posterior means with 95% highest posterior density credible intervals (CI). For further details, see “*Statistical analysis*” in the Supporting Information, as well as Tables S1–S3 for full model output.

## Results and Discussion

### Pharmaceutical Concentrations in the Brain

**Figure 2:**
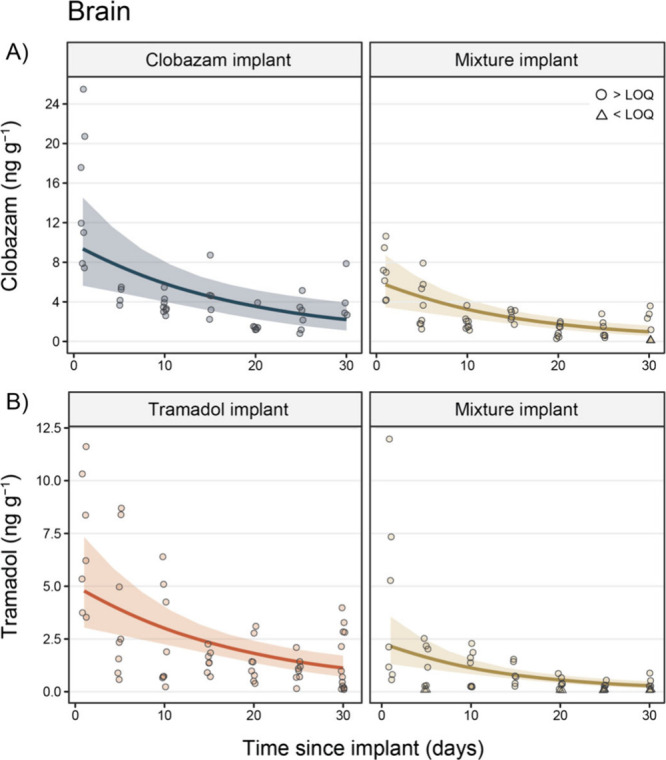
Concentrations (ng g^–1^) of (A) clobazam and (B) tramadol in the brains of fish in the clobazam (blue), tramadol (orange), and mixture (gold) implant treatment groups. Circle data points are those that were above the limit of quantification (LOQ), while triangle points indicate observations < LOQ (mean LOQ ± *SE*; clobazam = 0.35 ± 0.02 ng g^–1^; tramadol = 0.17 ± 0.01 ng g^–1^). Trend lines display the marginal mean concentration of each pharmaceutical over time (after controlling for year) extracted from the Bayesian generalized linear models, while colored ribbons denote 95% credible intervals. Note: points have been slightly jittered around the *x*-axis to aid visualization.

Clobazam concentrations peaked in the brain 1 day after implanting in both the clobazam implant (mean ± *SE* = 14.58 ± 2.59 ng g^–1^) and mixture implant (6.96 ± 0.93 ng g^–1^) treatment groups, decreasing over time in both groups to 4.33 ± 1.21 ng g^–1^ and 1.46 ± 0.55 ng g^–1^ at 30 days after implanting in both groups, respectively (Figure 2; Tables S1 and S4). After accounting for year and days since implantation, clobazam concentrations in the brain were, on average, 1.93 (95% CI = 1.16, 2.79) times greater in the clobazam-implant group compared to the mixture-implant group.

Tramadol concentrations also peaked in the brain 1 day after implanting in both the tramadol implant (7.02 ± 1.20 ng g^–1^) and mixture implant (4.18 ± 1.62 ng g^–1^) treatment groups, decreasing over time to 1.39 ± 0.39 ng g^–1^ and 0.318 ± 0.11 ng g^–1^ at 30 days after implanting in both groups, respectively (Figure 2; Tables S1 and S4). After accounting for year and days since implantation, tramadol concentrations were, on average, 3.14 (95% CI = 2.03, 4.34) times greater in the brains of the tramadol-implant group when compared to the mixture-implant group.

### Pharmaceutical Concentrations in the Muscle

**Figure 3:**
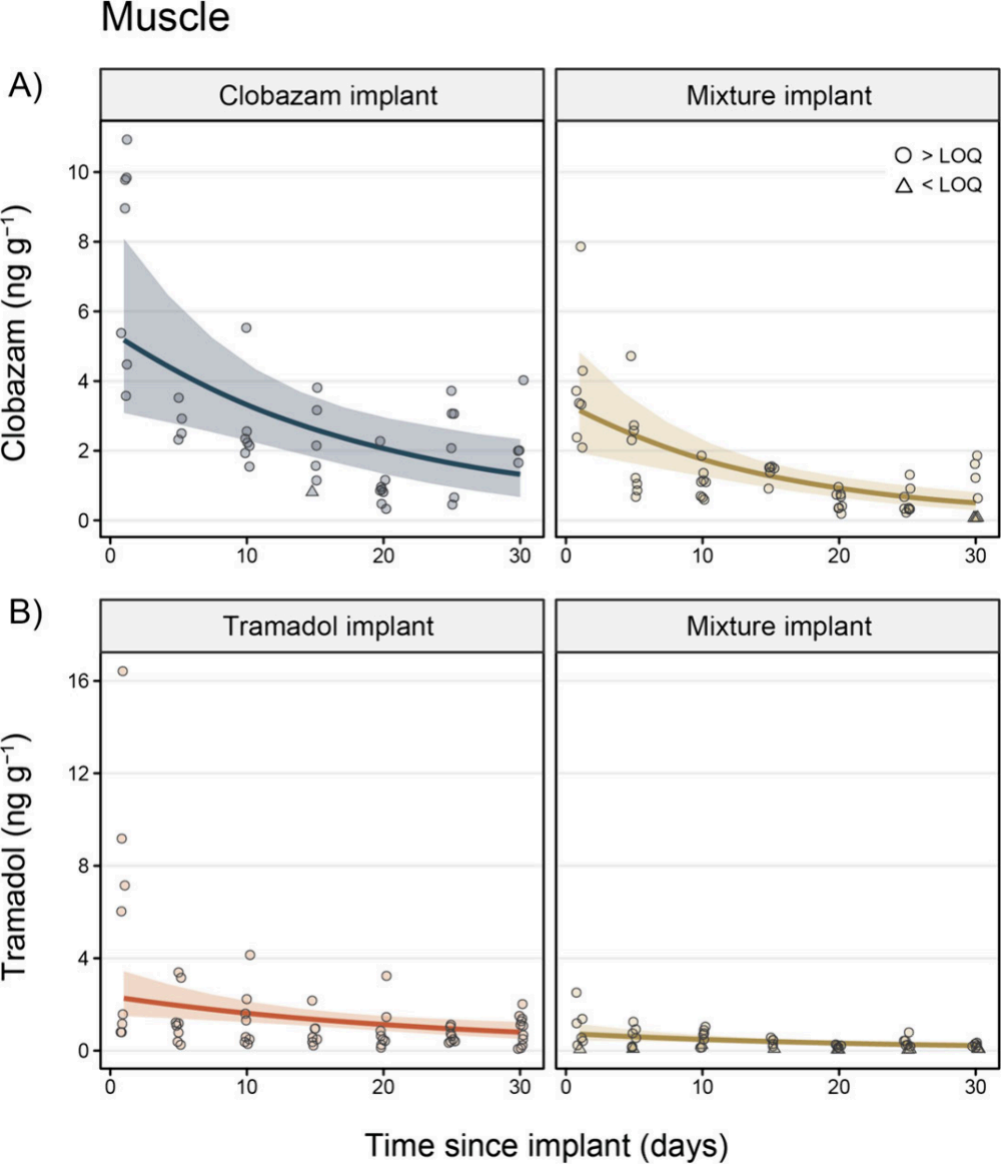
Concentrations (ng g^–1^) of (A) clobazam and (B) tramadol in the muscle of fish in the clobazam implant (blue), tramadol implant (orange), and mixture implant (gold) treatment groups. Circle data points are those that were above the limit of quantification (LOQ), while triangle points indicate observations < LOQ (mean LOQ ± *SE*; clobazam = 0.35 ± 0.02 ng g^–1^; tramadol = 0.17 ± 0.01 ng g^–1^). Trend lines display the marginal mean concentration of each pharmaceutical over time (after controlling for year) extracted from the Bayesian generalized linear models, while colored ribbons denote 95% credible intervals. Note: points have been slightly jittered around the *x*-axis to aid visualization.

Similar to the brain, clobazam concentrations in muscle samples peaked in the clobazam implant group 1 day after implanting (7.56 ± 1.13 ng g^–1^) and decreased over time (day 30 = 2.42 ± 0.54 ng g^–1^). This was the same for the mixture implant group, where clobazam concentrations in the muscle peaked 1 day after implanting (3.86 ± 0.73 ng g^–1^) and steadily decreased until the end of the experiment (day 30 = 0.79 ± 0.29 ng g^–1^; Figure 3; Tables S2 and S5). After accounting for year and days since implantation, clobazam concentrations were 2.08 (95% CI = 1.30, 2.99) times greater in the muscles of clobazam-implant fish when compared to those exposed via the mixture implant.

Tramadol concentrations also peaked 1 day after implanting in the muscles of tramadol implant (5.39 ± 1.96 ng g^–1^) and mixture implant (0.91 ± 0.32 ng g^–1^) treatment groups. These concentrations decreased over time in both the tramadol (day 30 = 0.91 ± 0.19 ng g^–1^) and mixture (day 30 = 0.20 ± 0.04 ng g^–1^) implant groups (Figure 3; Tables S2 and S5). Tramadol concentrations were also, on average, 3.42 (95% CI = 2.30, 4.77) times greater in the muscle of the tramadol-implant group when compared to the mixture-implant group (after accounting for year and days since implantation).

### Pharmaceutical Concentrations in the Liver

**Figure 4:**
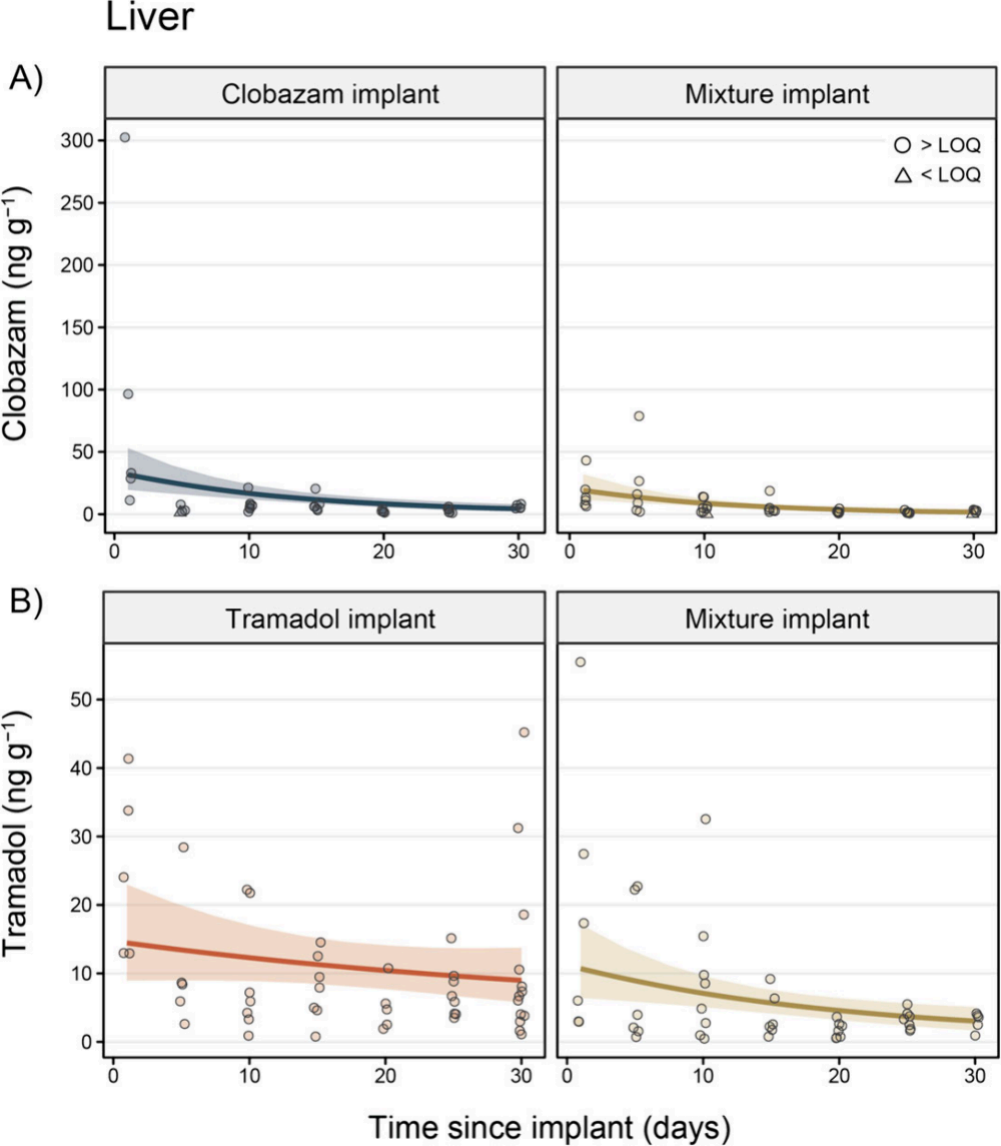
Concentrations (ng g^–1^) of (A) clobazam and (B) tramadol in the livers of fish in the clobazam implant (blue), tramadol implant (orange), and mixture implant (gold) treatment groups. Circle data points are those that were above the limit of quantification (LOQ), while triangle points indicate observations < LOQ (mean LOQ ± *SE*; clobazam = 0.35 ± 0.02 ng g^–1^; tramadol = 0.17 ± 0.01 ng g^–1^). Trend lines display the marginal mean concentration of each pharmaceutical over time (after controlling for year) extracted from the Bayesian generalized linear models, while colored ribbons denote 95% credible intervals. Note: points have been slightly jittered around the *x*-axis to aid visualization.

When comparing all tissues, pharmaceutical concentrations were highest in the liver of exposed fish. Specifically, clobazam concentrations peaked in the liver 1 day after exposure in the clobazam implant group (94.37 ± 54.00 ng g^–1^) and quickly declined over time (day 30 = 5.98 ± 0.73 ng g^–1^; Figure 4; Tables S3 and S6). Liver clobazam concentrations were, on average, 2.07 (95% CI = 1.24, 3.07) times greater in the clobazam implant group when compared to the mixture implant groups (after controlling for year and days since implantation), whereby concentrations peaked 1 day after exposure at 16.87 ± 5.60 ng g^–1^ and decreased to 2.25 ± 0.65 ng g^–1^ at 30 days post-implantation in the mixture implant group.

Similarly, tramadol concentrations in the liver peaked 1 day after implantation in the tramadol implant group (25.02 ± 5.64 ng g^–1^) and the mixture implant group (18.71 ± 8.34 ng g^–1^; Figure 4; Tables S3 and S6). These concentrations moderately decreased over time in the tramadol implant (day 30 = 11.33 ± 3.63 ng g^–1^) and mixture implant (day 30 = 3.00 ± 0.59 ng g^–1^) groups (Figure 4). Regardless of days since implantation or year, liver concentrations of tramadol were, on average, 2.28 (95% CI = 1.43, 3.25) times greater in the tramadol implant group when compared to the mixture implant group (Figure 4).

## Conclusions

Taken together, our results suggest that slow-release implants are an effective method for manipulating pharmaceutical exposure in Atlantic salmon smolts. This method is both highly cost-effective (∼$1.10 USD per fish) and valuable for field-based ecotoxicology experiments, allowing remote exposure of fish—and other aquatic species—to pharmaceuticals and their mixtures. When combined with field-sampling or biologging techniques,^13^ slow-release implants enable researchers to monitor species’ responses to controlled concentrations of chemical contaminants under real-world conditions. We found that concentrations of both clobazam and tramadol peaked in all tissues at 1 day after the administration of slow-release implants, after which both drugs reached relatively stable, slowly declining concentrations for the remainder of the 30-day sampling period. The highest concentrations of both drugs were detected in the liver, followed by the brain and then muscle. Importantly, average tissue concentrations found in the current study are broadly similar to those reported for opioid analgesics and benzodiazepine drugs found in the tissues of fish from exposed systems in the wild, where reported concentrations are typically in the low (e.g., <10) ng g^–1^ range.^34,41^ Further, observed concentrations were found to be higher in the single-exposure treatments relative to the mixture exposure, a result that has also been demonstrated after waterborne exposure of European perch (*Perca fluviatilis*) to benzodiazepine drugs (with the exception of oxazepam^42^). Understanding the specific mechanism(s) for these lower tissue concentrations in mixture-exposed fish requires further research, although this phenomenon is potentially due to constrained diffusion of drug mixtures from implants based on the total available implant surface area and/or competitive inhibition at drug transporters.^43^ This presents a complication when exposing to mixtures, given that results must be interpreted in the context of lower API accumulation in mixture treatments relative to individual-API treatments (an issue that can be overcome with pilot studies and analytical verification of tissue concentrations). An additional interesting avenue for future research, given that salmon in this study were assessed at the same development stage, is to investigate whether and how the smoltification process alters pharmaceutical kinetics (absorption, distribution, metabolism, and elimination).^44^ Furthermore, in this study, we were unable to identify the sex of smolts prior to implantation (as they have no external sex-determining characteristics at this age) or during dissections (because they are juveniles with no visible gonad development). However, potential sex differences in chemical accumulation will be an important consideration for future work in this area, as sex-based differences in contaminant uptake from environmental matrices have been noted.^45^ Overall, slow-release pharmaceutical implants provide a highly useful and controlled method of administering APIs to aquatic species in field-based ecotoxicology research, which promises to greatly broaden our understanding of the impacts of APIs on wildlife living in an increasingly polluted world.

## Data Availability

All data and R scripts associated with this paper are available from the Open Science Framework repository. Web link: https://osf.io/v82w9/ DOI: 10.17605/OSF.IO/V82W9.

## Supporting Information Available

Additional information on the study species, sample preparation and analysis, and statistical analysis, as well as Tables S1–S7. (PDF)
