# Supplementary material for: Slow-Release Pharmaceutical Implants in Ecotoxicology: Validating Functionality across Exposure Scenarios
Source: ACS Environ Au. 2024 Nov 25;5(1):69–75. doi: 10.1021/acsenvironau.4c00056 (PMC11741056; doi:10.1021/acsenvironau.4c00056)
Supplement: Supplementary file 1 — vg4c00056_si_001.pdf [file vg4c00056_si_001.pdf]

Supporting Information: Slow-release pharmaceutical implants in ecotoxicology: validating functionality across exposure scenarios

Michael G. Bertram[1,2,3,*], Jack A. Brand[1,4], Eli S.J. Thoré[1,5,6], Daniel Cerveny[1,7], Erin S. McCallum[1], Marcus Michelangeli[1,8], Jake M. Martin[1,3,9], Jerker Fick[10], Tomas Brodin[1]

[1] *Department of Wildlife, Fish, and Environmental Studies, Swedish University of Agricultural Sciences, Umeå SE-907 36, Sweden*

[2] *Department of Zoology, Stockholm University, Stockholm 114 18, Sweden*

[3] *School of Biological Sciences, Monash University, Melbourne, 3800, Australia*

[4] *Institute of Zoology, Zoological Society of London, London NW1 4RY, United Kingdom*

[5] *TRANSfarm - Science, Engineering, & Technology Group, KU Leuven, Lovenjoel 3360, Belgium*

[6] *Laboratory of Adaptive Biodynamics, Research Unit of Environmental and Evolutionary Biology, Institute of Life, Earth, and Environment, University of Namur, Namur 5000, Belgium*

[7] *Faculty of Fisheries and Protection of Waters, South Bohemian Research Center of Aquaculture and Biodiversity of Hydrocenoses, University of South Bohemia in Ceske Budejovice, Vodnany 389 25, Czech Republic*

[8] *Australian Rivers Institute, Griffith University, Nathan 4111, Australia*

[9] *School of Life and Environmental Sciences, Deakin University, Waurn Ponds 3216, Australia*

[10] *Department of Chemistry, Umeå University, Umeå 907 36, Sweden*

[*]Author for correspondence (E-mail: michael.bertram@slu.se; Tel.: +46 (0)70 446 90 55).

Study species

Two-year-old Atlantic salmon smolts ($n = 256$) were randomly selected from hatchery stocks raised at the Fisheries Research Station of SLU Aqua (Älvkarleby, Sweden) to receive slow-release implants in both 2020 and 2021. Fish were kept in large flow-through tanks (1 m length × 1 m width × 0.3 m height; ~300 L; 2 tanks per treatment; 16 fish per tank), and were not fed during the exposure period, in line with standard husbandry practices for pre-migration Atlantic salmon smolts. Fish were maintained in flowing water sourced directly from the River Dal to simulate as closely as possible natural water conditions, such as water chemistry and temperature (mean ± SE water temperature during the study period: 2020 = 10.06 ± 0.28 °C; 2021 = 12.78 ± 0.47 °C). The River Dal (mean pH = 6.85; range = 6.41–7.89) is a ~555 km long river located in central Sweden that flows through a series of small towns and agricultural lands before eventually discharging into the Baltic Sea.[1] As the river is heavily regulated for hydropower and contains historic populations of Atlantic salmon, supplemental stocking of salmonid smolts is carried out annually in the river to bolster local populations.

Sample preparation and analysis

Implants were positioned in the ventral posterior portion of the intraperitoneal (IP) cavity. We first dissected muscle tissue (expected to have the lowest overall pharmaceutical concentration), followed by brain tissue. Both of these tissues can be collected without puncturing the IP cavity, as the dorsal flank is where the majority of the muscle is positioned in this species. The liver in salmonids is positioned within the IP cavity, but at the ventral anterior portion of the body, close to the heart. To minimise potential contamination by the implant, we dissected out the liver by making an incision and flap behind the gills and excised the lobe of the liver from there. All dissection tools were cleaned in ethanol between each fish.

To measure clobazam and/or tramadol concentrations in fish tissues, we weighed each tissue sample in a 2 mL polypropylene (PP) tube. Then, we added 50 ng of each internal standard (oxazepam-d5, CAS: 65854-78-6, and tramadol 13C-d3, CAS: 1261398-09-7; Merck) and added 1.5 mL of acetonitrile (CAS: 75-05-08, HPLC grade; Merck). Each sample was then homogenised for 4 min at 42,000 rpm with zirconium beads using a Mini Beadbeater (Biospec, Bartlesville, OK) and centrifuged at 17,500 g for 10 min using a Beckman Coulter Microfuge 22R Centrifuge. Afterwards, the supernatant was transferred into a 12 mL glass vial. We repeated this extraction process by adding another 1.5 mL of acetonitrile to the original PP tube,

combining the supernatants from both extractions. These combined extracts were then fully evaporated before being reconstituted using 150 μL of methanol (CAS: 67-56-1, LC-MS grade; Merck). After, the extract was transferred into a glass autosampler vial with a 200 μL insert and stored at –18 °C for at least 24 h prior to analysis to allow protein precipitation.

Before analysis, the sample was thawed and centrifuged for proteins and other solid particles to settle. All samples were analysed through liquid chromatography-tandem mass spectrometry (LC-MS/MS). We performed liquid chromatography using a C18 phase Hypersil gold column (50 × 2.1 mm ID × 3 μm particles, Thermo Fisher Scientific, San Jose, CA, USA) to separate the target analytes before mass spectrometry analysis (Table S7). We used ultrapure water (prepared in-house with a Millipore purification system; Merck) and LC-MS-grade methanol as mobile phase, both acidified with 0.1% formic acid (CAS: 64-18-6, 98–100% purity; Merck). Mass spectrometry was then performed using a triple stage quadrupole mass spectrometer (TSQ Quantiva, Thermo Scientific, San Jose, CA) equipped with a heated-electrospray ionisation (HESI) ion source. The mass spectrometer was coupled to an Accela LC pump (Thermo Fisher Scientific, San Jose, CA) and a PAL HTC autosampler (CTC Analytics AG, Zwingen, Switzerland).

Procedural blanks were prepared each day of the tissue extractions following the same protocol as the tissue samples, including spiking the internal standards. These were run once to rule out the possibility of contamination during the extraction. Besides these, injection of both mobile phases was performed after every 10th sample to check for possible carry-over effects. Neither clobazam or tramadol were detected in procedural blanks or injected mobile phase samples.

Linearity, precision, and limit of quantification (LOQ), were assessed as Quality assurance and quality control (QA/QC) measures, alongside measurement of blank samples. Quantification of target compounds was accomplished using the method of isotopic dilution (the internal standard approach). The instrumental LOQ was derived from a six-point standard curve ranging from 0.1 to 50 ng g^{-1}. Samples with concentrations below the LOQ were assigned half the relevant LOQ (mean LOQ ± *SE*; clobazam = 0.35 ± 0.02 ng g^{-1}; tramadol = 0.17 ± 0.01 ng g^{-1}) for inclusion in mean concentration calculations, consistent with established protocols.[2,3]

Statistical analysis

Prior to analysis, data cleaning (e.g. removing samples where the implant was actively touching the target tissue during sampling, samples damaged during preparation) was conducted to ensure high-quality data and resulted in tissues from a total of 182 fish being included in the analysis. Concentration data were analysed using Bayesian generalised linear models with an exponential distribution (log link) in the *brms*[4] package within the R statistical environment.[5] Posthoc comparisons were performed using the *modelbased*[6] and *emmeans*[7] packages and are reported as ratios of geometric means. All models were run on four chains with weakly informative priors for 3000 iterations (1000 warmup). For each model, the tissue (brain, muscle, liver) concentration (ng g^{-1}) of either clobazam or tramadol was included as a response variable, while treatment (clobazam implant, tramadol implant, or mixture implant), days since implant (1–30), year (2020 and 2021), as well as an interaction between treatment and days since implant, as well as treatment and year were included as fixed-effects. Year was centred (2020 = –0.5; 2021 = 0.5) and days since implant was left-centred (day 1 = 0) to facilitate model fitting and interpretation of model parameters. Posterior predictive checks were performed to ensure adequate model fits and all models converged with low among-chain variability ($\hat{R}$= 1.00). We report posterior means with 95% highest posterior density credible intervals (CI). See Tables S1–S3 for full model output. No clobazam or tramadol were detected in any fish from the control implant group.

Supplementary tables

Table S1. Mean estimates [95% CI] extracted from Bayesian generalised linear models of clobazam and tramadol concentrations in the brains of exposed fish. Note: estimates are reported on the link scale.

Pharmaceutical	Model parameter	Mean estimate [95 % CI]
Clobazam	Intercept	2.20 [1.74, 2.70]
	Treatment (mixture)	−0.49 [−1.13, 0.17]
	Days since implant	−0.05 [−0.08, −0.02]
	Year	−0.32 [−0.97, 0.32]
	Treatment (mixture):Days since implant	−0.01 [−0.05, 0.03]
	Treatment (mixture):Year	0.01 [−0.81, 0.83]
Tramadol	Intercept	1.53 [1.10, 2.00]
	Treatment (mixture)	−0.82 [−1.49, −0.17]
	Days since implant	−0.05 [−0.08, −0.03]
	Year	0.05 [−0.50, 0.58]
	Treatment (mixture):Days since implant	−0.02 [−0.06, 0.02]
	Treatment (mixture):Year	0.67 [−0.11, 1.47]

Table S2. Mean estimates [95% CI] extracted from Bayesian generalised linear models of clobazam and tramadol concentrations in the muscle of exposed fish. Note: estimates are reported on the link scale.

Pharmaceutical	Model parameter	Mean estimate [95 % CI]
Clobazam	Intercept	1.60 [1.15, 2.11]
	Treatment (mixture)	−0.49 [−1.12, 0.15]
	Days since implant	−0.05 [−0.08, -0.02]
	Year	−0.38 [−1.01, 0.25]
	Treatment (mixture):Days since implant	−0.02 [−0.05, 0.02]
	Treatment (mixture):Year	0.09 [−0.73, 0.90]
Tramadol	Intercept	0.76 [0.35, 1.20]
	Treatment (mixture)	−1.14 [−1.76, −0.51]
	Days since implant	−0.04 [−0.06, −0.01]
	Year	0.83 [0.29, 1.35]
	Treatment (mixture):Days since implant	−0.01 [−0.04, 0.03]
	Treatment (mixture):Year	−0.25 [−0.97, 0.50]

Table S3. Mean estimates [95% CI] extracted from Bayesian generalised linear models of clobazam and tramadol concentrations in the livers of exposed fish. Note: estimates are reported on the link scale.

Pharmaceutical	Model parameter	Mean estimate [95 % CI]
Clobazam	Intercept	3.40 [2.92, 3.95]
	Treatment (mixture)	−0.49 [−1.16, 0.21]
	Days since implant	−0.07 [−0.10, −0.04]
	Year	0.69 [−0.06, 1.41]
	Treatment (mixture):Days since implant	−0.01 [−0.06, 0.03]
	Treatment (mixture):Year	−0.28 [−1.16, 0.63]
Tramadol	Intercept	2.64 [2.19, 3.13]
	Treatment (mixture)	−0.37 [−1.02, 0.30]
	Days since implant	−0.02 [−0.04, 0.01]
	Year	0.05 [−0.48, 0.59]
	Treatment (mixture):Days since implant	−0.03 [−0.07, 0.01]
	Treatment (mixture):Year	1.15 [0.36, 1.94]

Table S4. Tissue concentration (ng g^{-1}) of chemicals measured in the brains of exposed fish. Mean (± SE) [number of samples >LOQ / total sample size]. All values are averaged across years. (LOQ = Limit of quantification).

Pharmaceutical (ng g^{-1})	Treatment	Days since implant						
		1	5	10	15	20	25	30
Clobazam	Clobazam	14.578 (2.588) [7/7]	4.645 (0.441) [4/4]	3.656 (0.319) [8/8]	4.686 (1.108) [5/5]	1.778 (0.431) [6/6]	2.650 (0.658) [6/6]	4.328 (1.208) [4/4]
	Mixture	6.964 (0.928) [7/7]	3.701 (0.850) [8/8]	1.929 (0.283) [8/8]	2.618 (0.241) [6/6]	1.111 (0.234) [8/8]	1.201 (0.339) [7/7]	1.455 (0.548) [4/7]
Tramadol	Tramadol	7.020 (1.197) [7/7]	3.740 (1.150) [8/8]	2.497 (0.847) [8/8]	1.447 (0.202) [7/7]	1.422 (0.358) [8/8]	1.044 (0.204) [8/8]	1.388 (0.392) [13/13]
	Mixture	4.181 (1.615) [7/7]	1.079 (0.364) [6/8]	1.018 (0.304) [8/8]	0.846 (0.216) [6/6]	0.362 (0.101) [6/8]	0.228 (0.065) [4/7]	0.318 (0.112) [4/7]

Table S5. Tissue concentration (ng g^{-1}) of chemicals in the muscle of exposed fish. Mean (± SE) [number of samples >LOQ / total sample size]. All values are averaged across years. (LOQ = Limit of quantification).

Pharmaceutical (ng g^{-1})	Treatment	Days since implant						
		1	5	10	15	20	25	30
Clobazam	Clobazam	7.562<br>(1.129)<br>[7/7]	2.815<br>(0.267)<br>[4/4]	2.612<br>(0.501)<br>[7/7]	2.108<br>(0.480)<br>[5/6]	0.971<br>(0.209)<br>[8/8]	2.173<br>(0.555)<br>[6/6]	2.421<br>(0.541)<br>[4/4]
	Mixture	3.864<br>(0.725)<br>[7/7]	2.017<br>(0.480)<br>[8/8]	1.065<br>(0.149)<br>[8/8]	1.396<br>(0.099)<br>[6/6]	0.555<br>(0.093)<br>[8/8]	0.557<br>(0.135)<br>[8/8]	0.792<br>(0.293)<br>[4/7]
Tramadol	Tramadol	5.391<br>(1.955)<br>[8/8]	1.433<br>(0.421)<br>[8/8]	1.381<br>(0.463)<br>[8/8]	0.822<br>(0.247)<br>[7/7]	0.938<br>(0.359)<br>[8/8]	0.648<br>(0.105)<br>[8/8]	0.909<br>(0.191)<br>[11/11]
	Mixture	0.909<br>(0.323)<br>[6/7]	0.505<br>(0.155)<br>[7/8]	0.550<br>(0.124)<br>[7/8]	0.342<br>(0.076)<br>[5/6]	0.166<br>(0.030)<br>[6/8]	0.306<br>(0.086)<br>[6/8]	0.198<br>(0.039)<br>[5/7]

Table S6. Tissue concentration (ng g^{-1}) of chemicals in the livers of exposed fish. Mean (± SE) [number of samples >LOQ / total sample size]. All values are averaged across years. (LOQ = Limit of quantification).

Pharmaceutical (ng g^{-1})	Treatment	Days since implant 1	5	10	15	20	25	30
Clobazam	Clobazam	94.365 (54.003) [5/5]	3.577 (1.404) [3/4]	7.962 (2.374) [7/7]	7.956 (2.599) [6/6]	2.286 (0.420) [6/6]	3.017 (0.824) [6/6]	5.981 (0.725) [5/5]
	Mixture	16.866 (5.604) [6/6]	22.586 (11.826) [6/6]	5.866 (1.890) [7/8]	5.742 (2.622) [6/6]	1.903 (0.475) [7/7]	1.510 (0.338) [7/7]	2.254 (0.645) [4/5]
Tramadol	Tramadol	25.022 (5.640) [5/5]	10.801 (4.537) [5/5]	9.363 (3.346) [7/7]	7.833 (1.812) [7/7]	5.106 (1.569) [5/5]	7.233 (1.379) [8/8]	11.330 (3.626) [13/13]
	Mixture	18.705 (8.340) [6/6]	8.876 (4.323) [6/6]	9.415 (3.752) [8/8]	3.811 (1.327) [6/6]	1.734 (0.449) [7/7]	3.235 (0.513) [7/7]	3.000 (0.588) [5/5]

Table S7. LC gradient for the separation of target compounds.

Time (min)	A, %	B, %	Flow, µL min $^{-1}$
0	98	2	250
1	98	2	250
4	2	98	250
6	2	98	250
6.01	98	2	250
7.5	98	2	250

A, water + 0.1% formic acid. B, Methanol + 0.1% formic acid.

References

(1) Beier, U. Heavily modified waters in Europe case study on the River Dalälven. National Board of Fisheries, Institute of Freshwater Research, Drottingholm. **2002**.

(2) Antweiler, R. C.; Taylor, H. E. Evaluation of statistical treatments of left-censored environmental data using coincident uncensored data sets: I. Summary statistics. *Environ. Sci. Technol.* **2008**, 42, 3732–3738.

(3) McCallum, E. S.; Cerveny, D.; Fick, J.; Brodin, T. Slow-release implants for manipulating contaminant exposures in aquatic wildlife: a new tool for field ecotoxicology. *Environ. Sci. Technol.* **2019**, 53, 8282–8290.

(4) Bürkner, P. C. *brms*: an R package for Bayesian multilevel models using Stan. *J. Stat. Softw.* **2017**, 80, 1–28.

(5) R Development Core Team. R: a language and environment for statistical computing. Vienna, Austria: R Foundation for Statistical Computing. **2019**.

(6) Lüdecke, D.; Ben-Shachar, M. S.; Patil, I.; Wiernik, B. M.; Bacher, E.; Thériault, R.; Makowski, D. *easystats*: framework for easy statistical modeling, visualization, and reporting. CRAN. https://easystats.github.io/easystats/. **2022**.

(7) Lenth, R. V.; Buerkner, P.; Giné-Vázquez, I.; Herve, M.; Jung, M.; Love, J.; Miguez, F.; Riebl, H.; Singmann, H. *emmeans*: estimated marginal means, aka least-squares means (R package version 1.8.2). **2022**.
